# Comparison and evaluation of stresses generated by rapid maxillary expansion and the implant-supported rapid maxillary expansion on the craniofacial structures using finite element method of stress analysis

**DOI:** 10.1186/s40510-016-0157-6

**Published:** 2017-01-16

**Authors:** Varun Jain, Tarulatha R. Shyagali, Prabhuraj Kambalyal, Yagnesh Rajpara, Jigar Doshi

**Affiliations:** 1 Department of Orthodontics and Dentofacial Orthopedics, Darshan Dental College and Hospital, Loyara, Udaipur, India; 2 Department of Orthodontics and Dentofacial Orthopeadics, Hitkarini Dental College and Hospital, Jabalpur, MP India; 3 Department of Orthodontics and Dentofacial Orthopedics, Goenka institute of dental sciences, Ahmedabad, India

**Keywords:** Rapid maxillary expansion, Implant-supported rapid maxillary expansion, Finite element method, Stress, Displacement

## Abstract

**Background:**

The study aimed to evaluate and compare the stress distribution and 3-dimensional displacements along the craniofacial sutures in between the Rapid maxillary Expansion (RME) and Implant supported RME (I-RME).

**Method﻿s:**

Finite element model of the skull and the implants were created using ANSYS software. The finite element model thus built composed of 537692 elements and 115694 nodes in RME model & 543078 elements and 117948 nodes with implants model. The forces were applied on the palatal surface of the posterior teeth to cause 5mm of transverse displacement on either side of the palatal halves, making it a total of 10mm. The stresses and the displacement values were obtained and interpreted.

**Results:**

Varying pattern of stress and the displacements with both positive and negative values were seen. The maximum displacement was seen in the case of plain RME model and that too at Pterygomaxillary suture and Mid-palatal suture in descending order. In the case of I-RME maximum displacement was seen at Zygomaticomaxillary suture followed by Pterygomaxillary suture. The displacements produced in all the three planes of space for the plain RME model were greater in comparison to the Implant Supported RME model. And the stresses remained high for all the sutures in case of an I-RME.

**Conclusions:**

There is a definite difference in the stress and the displacement pattern produced by RME and I-RME model and each can be used according to the need of the patient. The stresses generated in case of conventional RME were considerably less than that of the I-RME for all the sutures.

## Background

The rapid maxillary expansion is the treatment of choice in cases of malocclusion involving the transverse maxillary deficiencies and the class III malocclusion. In case of transverse maxillary deficiency, the orthopedic forces of rapid maxillary expansion will bring about the dental as well as the skeletal expansion of the narrow maxilla to fetch the space for relieving of the crowding or the proclination or to level the bite [1, 2] and it also increases the nasal permeability and nasal width and straightens the nasal septum [3–5].

Whereas, in class III malocclusion cases, the loosening of the circumzygomatic sutures will make the maxilla pliable enough to respond to the orthopedic protrusive forces of the protraction face mask [6]. All the above said changes are applicable to the patients who are growing, and the adult patients who require the similar changes have to undergo surgically assisted rapid maxillary expansion [7] procedure, which is quite invasive. The alternative is to go ahead with the ankylosed tooth as a support [8] or else to utilize the osteosynthesis plates for expansion. But these have their own set of disadvantages like invasive operation, with a higher risk of infection and speech problems as the appliance limits the tongue movement [9, 10]. Apart from this, the traditional RME appliances at certain times are bound to produce the side effects like root resorption, bony dehiscence, and decreases in the thickness of the buccal cortical plate, undesirable tooth movements, and relapse and loss of buccal cortical bone at the anchorage teeth.

As a replacement, we can utilize the properties of orthodontic implants to apply the force on the palatal shelves through the medium of appliance to obtain the orthopedic changes and such appliance are known as implant-supported rapid maxillary expansion appliances (I-RME). These appliances apply the force directly on to the implant embedded in the bone, thus overcoming the disadvantages of the earlier appliances. As they are anchored to the palate, it is anticipated that a more efficient skeletal expansion and decreased undesired dental effects are produced [11–15].

The literature pertaining to the impact of rapid maxillary expansion on different circumzygomatic bones is only limited to the traditional appliances, and there are very few articles which have explored the possibilities of the implant-supported RMEs [11]. Different designs of micro-implanted supports for anchorage control are different from one study to the other. Thus, the current study plans to compare the effects of the traditional RME with that of the implant-supported RME using the finite element method of the stress analysis. The finite element analysis (FEA) has proven its worth in the field of orthodontics since long [11, 15–20], and the present study utilizes FEA’s ability of virtual model construction and the stresses analysis with the hypothesis that the implant-supported RME produces the similar effects as that of the simple RME on the different craniofacial sutures.

## Methods

Initial step in the creation of the finite element model of the skull involved the obtaining of the CT scan images of the skull of the 12-year-old boy using an X-Force/SH spiral CT scan machine (manufactured by Toshiba, Japan). The CT scan sections were obtained from DICOM images (Digital Imaging and Communication of Medicine). The CT section were obtained at the interval of 2.5 mm intervals in the parallel horizontal planes as the obtained images at this interval were capable producing better geometric models [8] than the models used in the previous studies [17, 19].

**Fig. 1:**
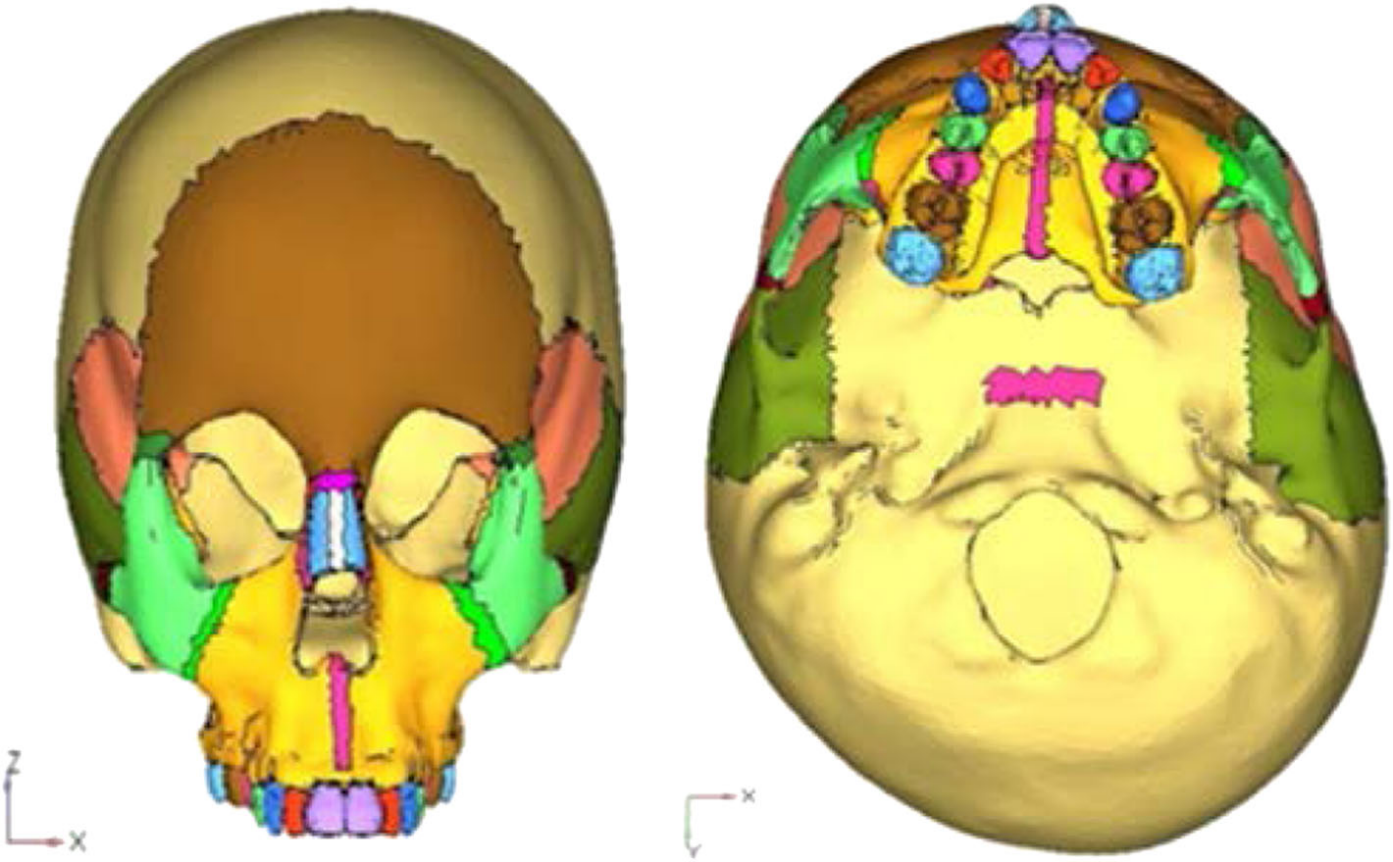
Geometric model of the skull

These DICOM images were then fed into the computer, and each layer created was stacked one above the other in the axial direction and joined by straight lines. Using the MIMICS (Materialise’s Interactive Medical Image Control System) software, these cross sections were converted into a three-dimensional mathematical model. Thus, a virtual geometric model of the skull was obtained (Fig. 1).

The implants were constructed using reverse-engineering process. Reverse engineering has become a viable method to create a three-dimensional virtual model of an existing physical part; it involves measuring an object and then reconstructing it as a three-dimensional model. Dentos implant design: SH1312-08 [AbsoAnchor, Dentos Inc, Daegu, Korea] i.e., 1.3 mm (diameter) × 8 mm (length) was modeled.

**Fig. 2:**
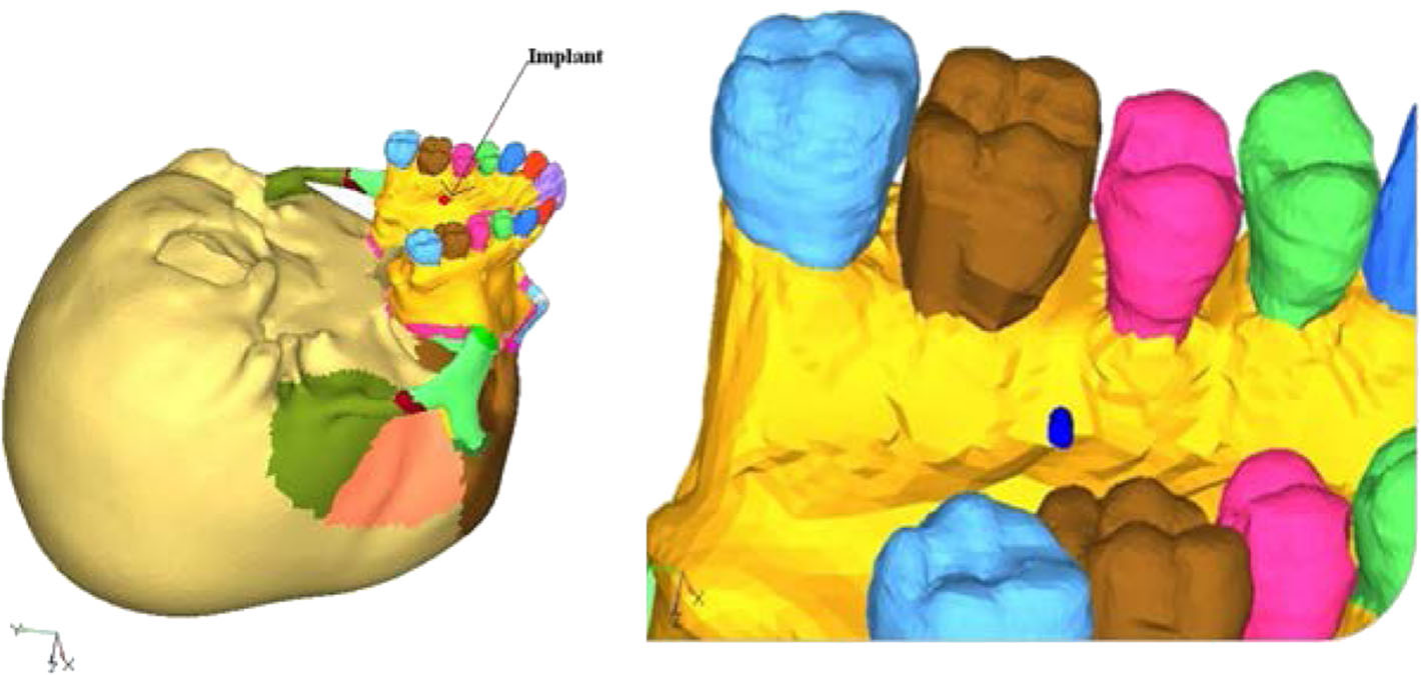
Geometric model with implant embedded in the bone

**Fig. 3:**
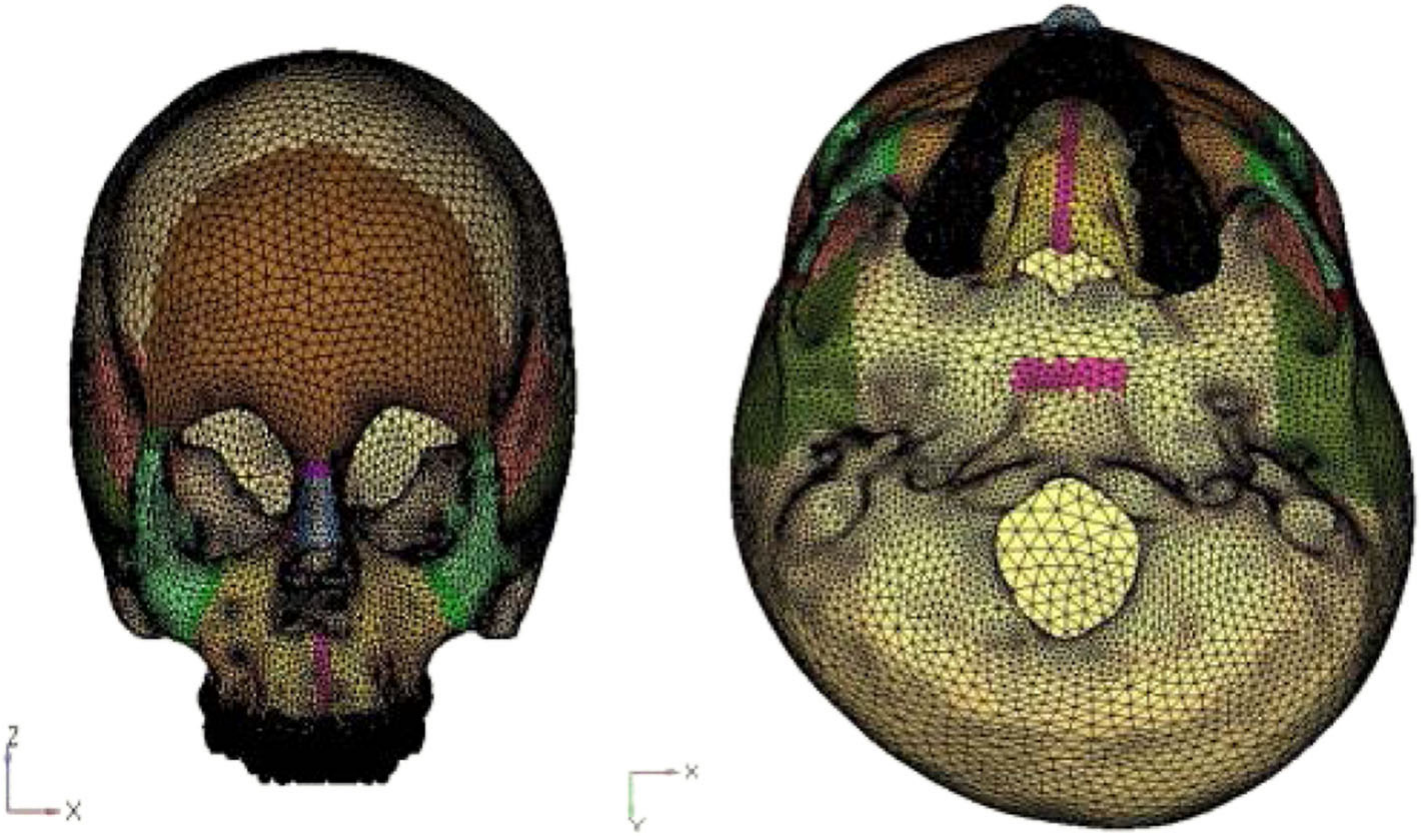
Finite element model of the skull

**Fig. 4:**
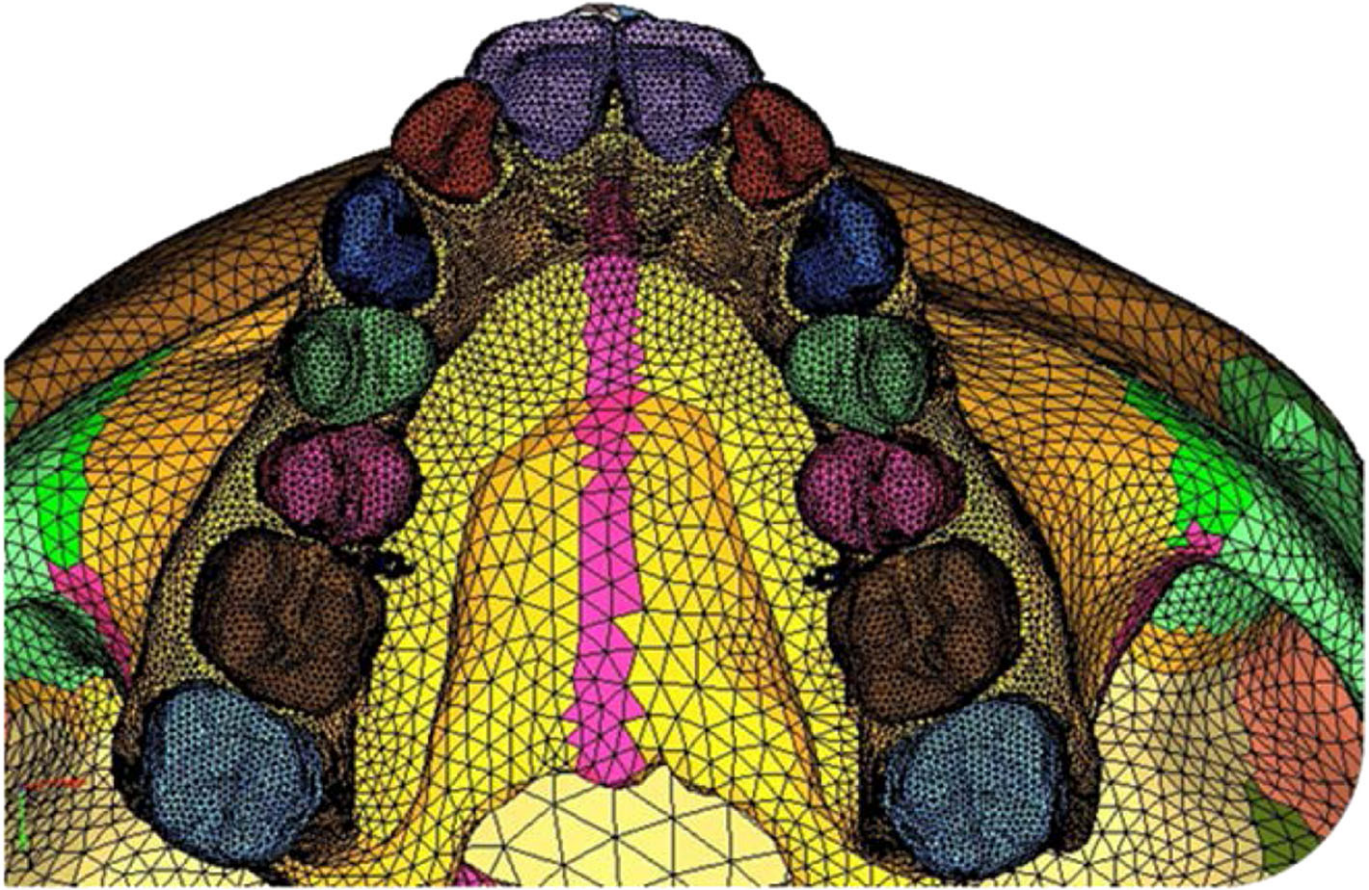
Finite element model of comprising of implant

The constructed implant was then embedded in the three-dimensional skull model at the desired site (Fig. 2). Next step involved the meshing of the geometric model using the finite element method. Two such mesh models were prepared, one with implant and the other without the implant. The mesh structure chosen was hyper mesh 0.7, which is a four-nodded tetrahedral element. ANSYS software was used to create the finite element model. The finite element model thus built comprised of 537,692 elements and 115,694 nodes in without implant model (Fig. 3) and 543,078 elements and 117,948 nodes with implants model (Fig. 4).

The constructed finite element model had nine sutures (midpalatal suture, naso-maxillary suture, zygomatico-maxillary suture, pterygo-maxillary suture, intranasal suture, fronto-maxillary suture, naso-frontal suture, zygomatico-temporal suture, zygomatico-frontal suture) and the spheno-occipital synchondrosis. The model allowed independent movement of the bones adjacent to the cranial sutures in response to the stimulated orthopedic forces.

The material properties for the compact bone, cancellous bone, tooth, sutures, spheno-occipital synchondrosis, and titanium (Table 1) were obtained from the previous published literature and were fed into the finite element model [17, 21, 22]. All the structures modeled were assumed to be isotropic and homogeneous.

**Table 1:** Mechanical properties of various materials

Material	Modulus of elasticity	Poisson’s ratio
Compact bone	1.37 × 10^3^	0.3
Cancellous bone	7.9 × 10^2^	0.3
Tooth	2.0 × 10^3^	0.3
Sutures	1.46 MPa	0.28
Cartilage	0.69 MPa	0.018
Titanium	114 MPa	0.34

**Fig. 5:**
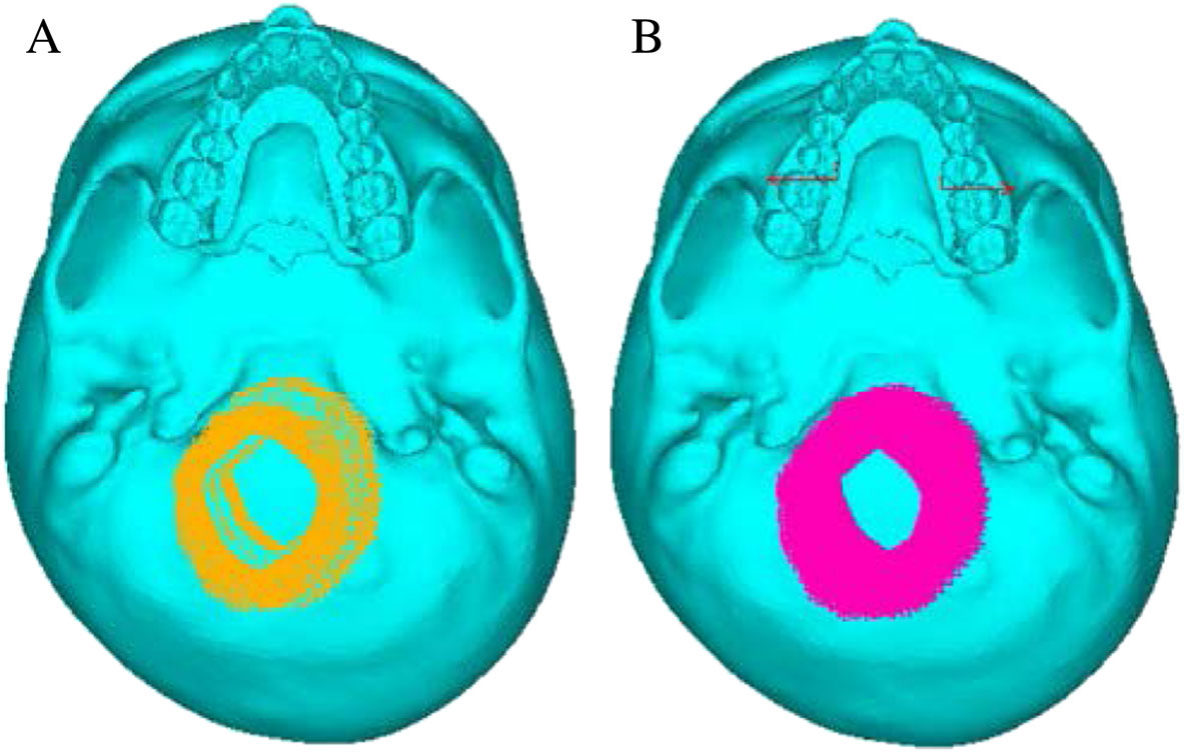
Boundary conditions of the finite element model. **a** Without implants. **b** With implants

**Fig. 6:**
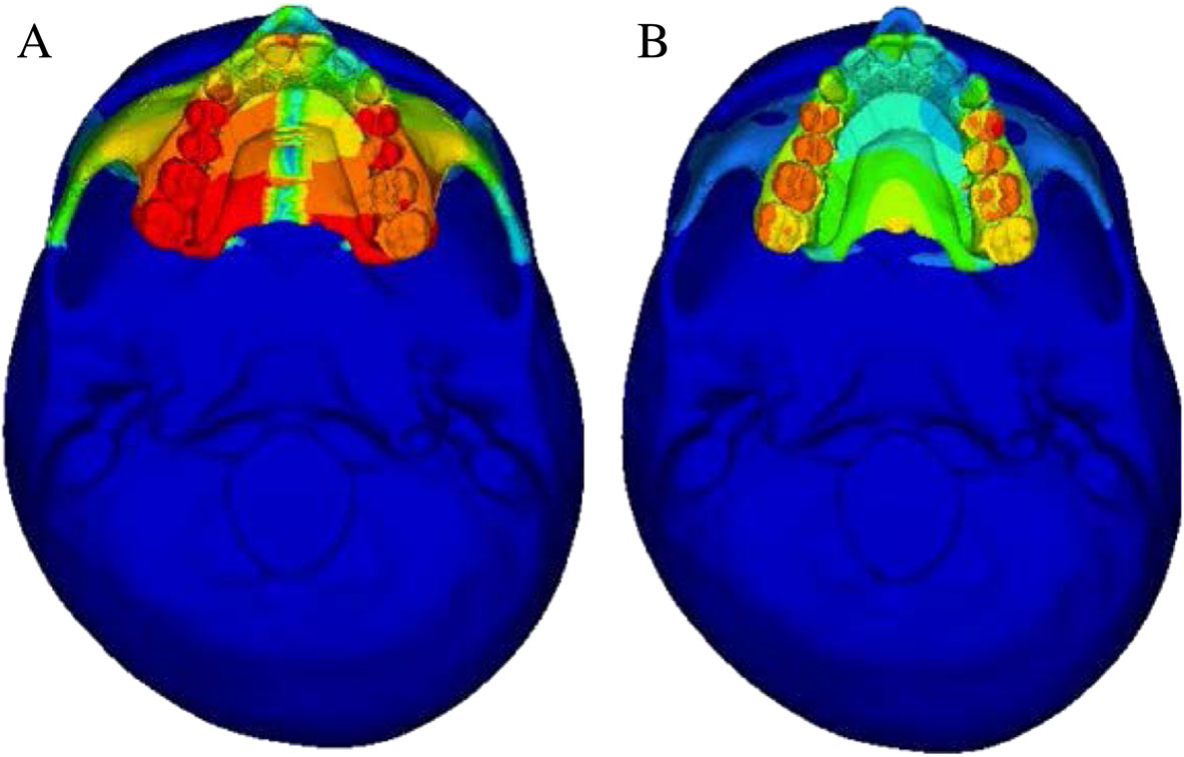
Overall skull view after application of the forces. **a** Without implants. **b** With implants

A zero-displacement and a zero-rotation boundary condition were imposed on the nodes along the foramen magnum (Fig. 5a, b). An orthopedic force of 102.32 N magnitude was applied on the maxillary premolar and first molar crown, in plain RME model and on the implants in case of implant-supported RME, which produced the total of 10 mm expansion (which equaled to 5 mm expansion on each side) on both the models (Fig. 6). The deflection and the von Mises stresses were studied using the ANSYS software.

## Results

**Fig. 7:**
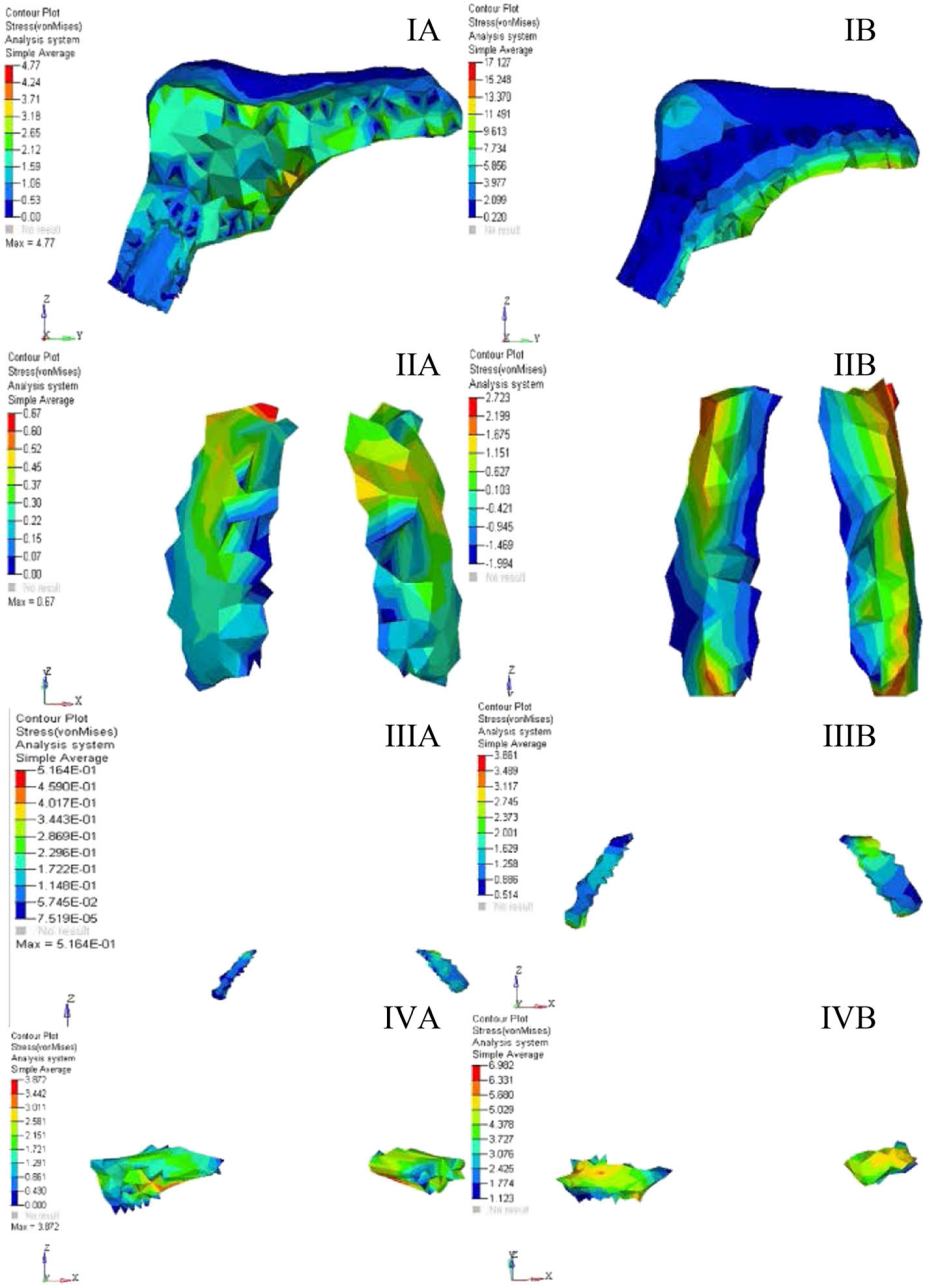
von Mises stresses at different sutures for **A** RME. **B** Implant-supported RME. **IA**, **IB** Midpalatal suture. **IIA**, **IIB** Naso-maxillary suture. **IIIA**, **IIIB** Zygomatico-maxillary suture. **IVA**, **IVB** Pterygo-maxillary suture

**Fig. 8:**
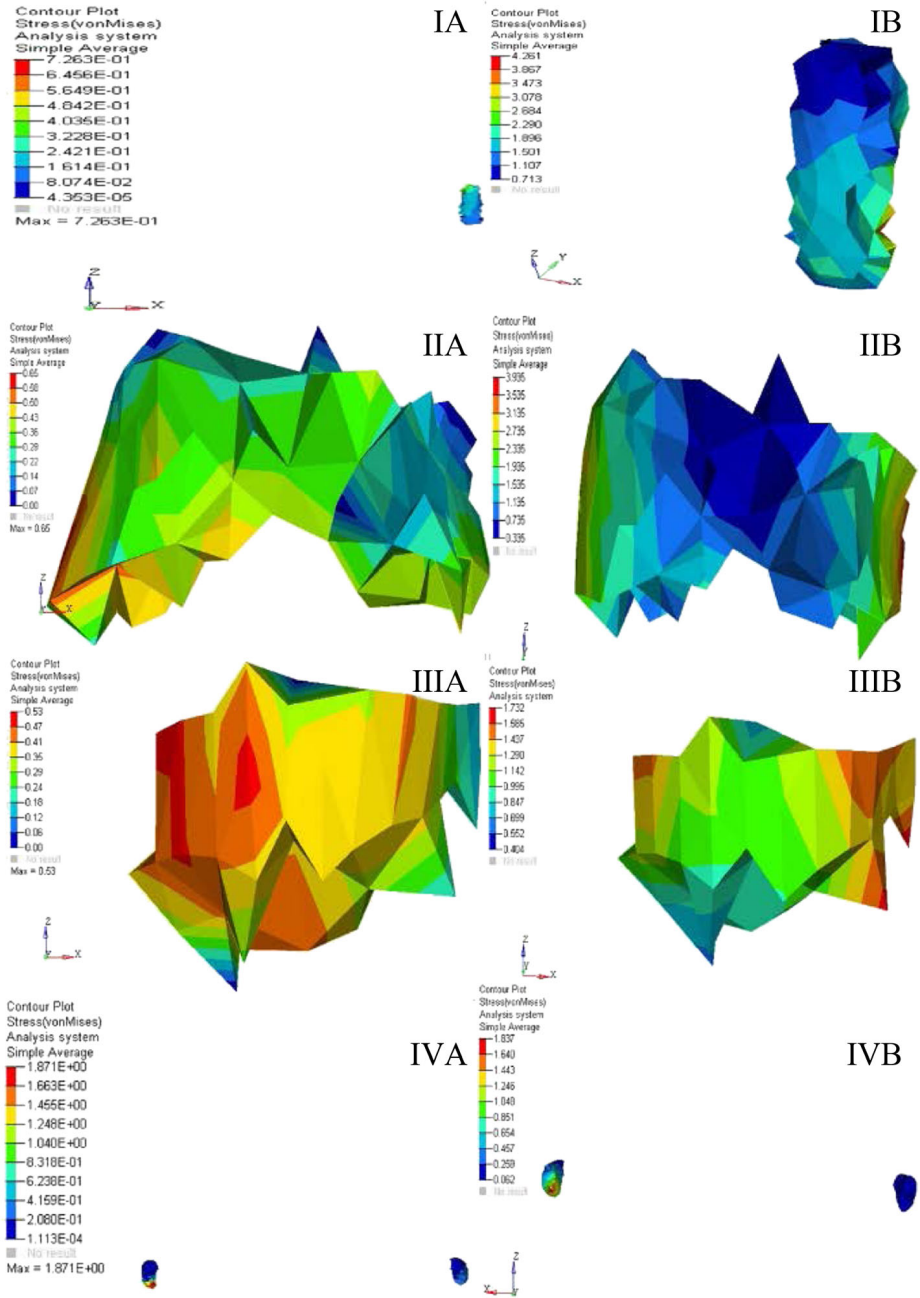
von Mises stresses at different sutures for **A** RME. **B** Implant-supported RME. **IA**, **IB** Intranasal suture. **IIA**, **IIB** Frontomaxillary suture. **IIIA**, **IIIB** Naso-frontal suture. **IVA**, **IVB** Zygomatico-temporal suture

**Fig. 9:**
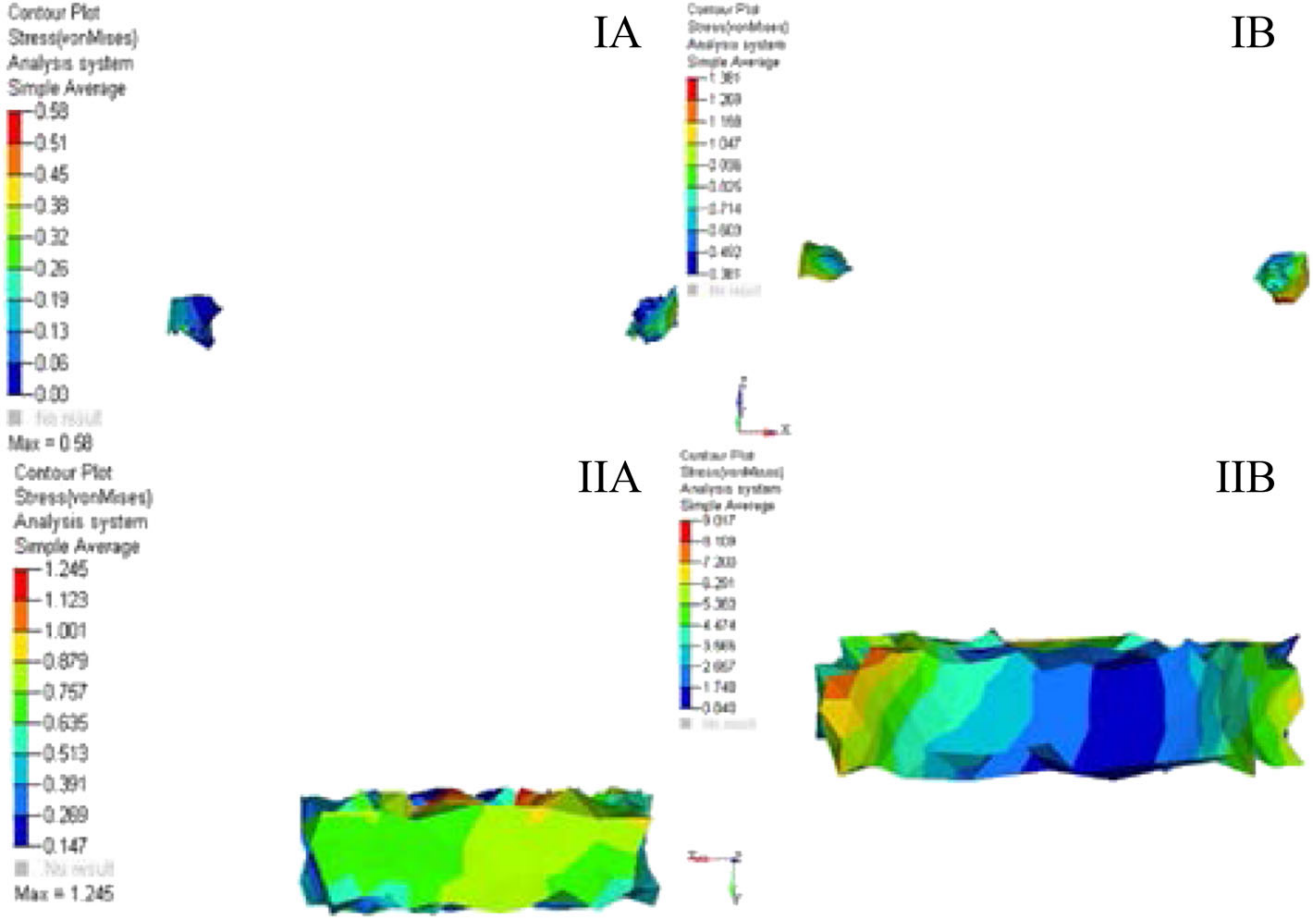
von Mises stresses at different sutures for **A** RME. **B** Implant-supported RME. **IA**, **IB** Zygomatico-frontal suture. **IIA**, **IIB** Spheno-occipital synchondrosis

The stress distribution was plotted using the general post processor of ANSYS. The stress distribution at different sutures is shown in Figs. 7, 8, and 9. The displacement pattern at the different suture sites for implant-supported RME and the standard RME is depicted in Table 2. Maximum amount of stress in case of implant-supported RME was noted in the midpalatal suture (17.12 MPa), followed by spheno-occipital synchondrosis (9.01 MPa), pterygo-maxillary suture (6.98 MPa), and the intranasal suture (4.26 MPa). Whereas, in the standard RME, maximum stresses were seen in the midpalatal suture (4.77 MPa). This is followed by pterygo-maxillary suture (3.87 MPa), zygomatico-temporal suture (1.87 MPa), and the spheno-occipital synchondrosis (1.24 MPa). The stress generated in the implant-supported RME was more in magnitude than the standard RME.

**Table 2:** Comparison of stresses between RME and I-RME

Site	RME		Implant-supported RME	
	Stress (MPa)	Principle stress contours (MPa)	Stress (MPa)	Principle stress contours (MPa)
Midpalatal suture	4.77	5.2	17.12	18.53
Naso-maxillary suture	0.67	0.62	2.72	2.52
Zygomatico-maxillary suture	0.51	0.53	3.86	3.37
Pterygo-maxillary suture	3.87	3.5	6.98	6.65
Intranasal suture	0.72	1.18	4.26	2.14
Fronto-maxillary suture	0.65	0.70	3.93	4.21
Naso-frontal suture	0.53	0.73	1.73	1.91
Zygomatico-temporal suture	1.87	0.74	1.83	1.80
Zygomatico-frontal suture	0.58	-0.21	1.38	0.86
Spheno-occipital synchondrosis	1.24	1.61	9.01	6.80

Table 3 shows the amount of displacement produced by the I-RME and the standard RME at different sutures. Displacement was noted for all the principle directions. Displacement for different sutures was more in case of plain RME than the implant-supported RME.

**Table 3:** Comparison of displacement between RME and I-RME

Site	RME			Implant-supported RME		
	“X” direction (mm)	“Y” direction (mm)	“Z” direction (mm)	“X” direction (mm)	“Y” direction (mm)	“Z” direction (mm)
Midpalatal suture	4.47	3.40	1.48	0.555	2.132	−1.242
Naso-maxillary suture	3.55	1.54	0.24	0.788	1.715	0.110
Zygomatico-maxillary suture	3.59	0.21	0.44	3.017	3.462	2.184
Pterygo-maxillary suture	4.64	1.01	0.58	1.119	2.127	1.612
Intranasal suture	0.392	1.80	−0.67	−0.72	2.28	0.045
Fronto-maxillary suture	1.53	1.18	0.03	0.12	2.39	0.45
Naso-frontal suture	0.54	1.14	0.001	−0.73	1.46	0.15
Zygomatico-temporal suture	0.78	0.49	1.38	−0.227	0.306	1.919
Zygomatico-frontal suture	0.19	0.68	1.09	0.43	1.02	1.57
Spheno-occipital synchondrosis	0.00002	0.002	0.0001	0.116	0.580	0.535

*X direction,* Negative value denotes lateral displacement; positive denotes medial displacement

*Y direction,* Negative value denotes posterior displacement; positive denotes anterior displacement

*Z direction,* Negative value denotes superior displacement; positive denotes inferior displacement

## Discussion

The biomechanical changes produced by the RME can be studied using various tools like conventional cephalometrics, strain gauge, photoelastic, or the halographic technique. The disadvantage of all these techniques is the failure to depict the results in three-dimensional spaces. Finite element method of stress analysis is the best method to check out the changes produced by the RME in three-dimensional space by the creation of virtual model and the possibilities of stimulating the clinical situation is innumerable with such techniques. [19] Thus, the present study utilized the benefits of the FEM to compare and analyze the difference between the traditional RME and the recently developed implant-supported RME.

In case of implant-supported RME, usually, the clinician places either four implants or two implants with the RME screw attached to it. And they can be tooth supported or completely bone supported. The placement of implant is in between two premolars bilaterally in case of two implants or else two implants in the anterior region and the two implants in the posterior region in case of four implant design [23, 24].

For this particular study, complete bone-supported design with the two implants was chosen. The implants were placed between the second premolar and the first molar area, as the region was predicted to be the safe zone for the placement of implants in the palatal region [25] and in few of the studies, it was the site of choice for the implant placement [26].

### Stresses and displacement pattern at the midpalatal suture 

The stresses generated in the case of plain RME were considerably less than that of the implant-supported RME for all the sutures. In both the models, the positive and the negative values were noted and these positive and negative values are indicative of the tensile and compressive stresses, respectively. The presence of differential strain pattern suggests the possibility of bone deposition and resorption at different parts of the same suture. Similar variation in the stress pattern was seen in the previous studies [18, 27]. The reason behind this differential stress could be answered through Newton’s first law, where it is stated that the application of force can change the state of rest. In case of the craniofacial bones, when the force is applied, they are displaced and the displacement of bones was not translator in nature as the force applied was not exactly at the center of resistance of a particular bone, thus the individual bones of the craniofacial region moved in different directions in three-dimensional view, producing positive and negative stress and strains at different locations of the same bone. Stresses at the midpalatal suture remained high in comparison to other suture in both RME and I-RME (Fig. 7IA, IB and Table 2). Higher stress concentration was seen on the posterior part of the midpalatal suture with the decreased stresses at the anterior segment for both the cases. Earlier literature also supported this finding [18, 28]. Reason behind the high stress on the midpalatal suture can be attributed to the vicinity of the applied force, which was nearer to the suture.

The transverse displacement pattern of the midpalatal suture in both the cases showed greater displacement of the palatal halves at the anterior section than the posterior section, indicating the fan-shaped opening of the suture, and the results were in accordance to the reports of the previous study [3, 17, 19, 29, 30]. However, in case of the I-RME, the opening of the posterior section was to a greater extent than the plain RME. Anterior and downward displacement of midpalatal suture was noted in case of RME, this was in accordance to the findings of the earlier studies on RME [3, 17, 19, 31, 32]. In case of I-RME, anterior and upward displacement was observed. Probably, the site of application of force away or nearer to the center of resistance of the bones is the reason for such different pattern of responses.

As stated in the previous studies, the separation of the sutures was pyramidal, with the base of the pyramid located at the oral side in the vertical plane and anteriorly along the antero-posterior plane for RME. Similar pattern of the opening was noticed in the present study. As the maxilla is attached to the sphenoid bone through the pterygo-maxillary fissure, this kind of pyramidal opening is bound to occur [33–35].

### Stress and displacement at the naso-maxillary suture

In one of the studies on the effects of RME, there was a significant increases in the width of the naso-maxillary suture and there was a difference of 0.4 mm in the pre- and posttreatment CT scans [30], same was true in our study as a maximum displacement of 3.55 mm was noted in the transverse plane in case of RME model (Fig. 7IIA, IIB and Table 2). Along with width increase, there was a generalized superior displacement, this finding was in accordance with the earlier studies on RME [31, 32, 36]. Width increase subsequently reduces the airflow resistance, which is one of the common clinical features in the patients with constricted maxilla. This orthopedic influence of the RME has been mentioned in the previous RME studies [37–40]. The displacement showed in I-RME was significantly less as compared to the RME model, indicating the inefficiency of implant-supported expansion. Conversely, greater amount of force may be required in the case of implant-supported RME to get similar results as plain RME.

The stresses generated by the models for the naso-maxillary suture were concentrated laterally toward the infra-orbital region. The results were in accordance to previous literature [18, 28]. However, the stresses generated by the I-RME models were greater than the RME model. The force applied by the RME and I-RME models was transverse in nature. Owing to this, all the sutures move away from the midline and it is not surprising to see the greater stress on the lateral wall of the naso-maxillary suture.

### Stress and displacement at the zygomatico-maxillary suture 

The zygomatico-maxillary suture in RME displaced laterally and posterosuperiorly, resulting in a wedge-shaped splitting of the maxilla along with a downward displacement thus, producing a similar displacement pattern on the zygomatic bone (Fig. 7IIIA, IIIB and Table 2). Contrasting results were seen in the study of Ghonemia et al. who showed an insignificant change in the width of the suture. However, there were studies which supported our results [17, 18]. Opposing effect was seen in the I-RME, with the suture rotating in postero-inferior direction thus, reducing the downward rotational movement of the maxilla.

The stresses generated in RME and I-RME showed positive and negative values, which indicate of the tensile and compressive stresses, respectively. Sutural growth is accelerated by both tension and compression with appropriate parameters such as strain amplitude, rate, and dose [41]. The presence of differential strain patterns suggests the possibility of differential bone remodeling along the same suture. Similar variation in the stress pattern was seen in the previous studies [18, 27]. Again, the stresses generated by I-RME remained high in comparison to the plain RME. In case of I-RME, the forces were directly applied on the implants embedded in the palate; as the palate is attached to different sutures, the impact of force will always be greater than the plain RME, where the forces are directed on the dentition.

### Stress and displacement at the pterygo-maxillary suture

The maximum displacement pattern at pterygo-maxillary suture in RME showed a medial (4.64 mm in the transverse plane), anterior (1.01 mm in the sagittal plane), and inferior (0.58 mm in the vertical plane) movement. Even in case of I-RME model, a similar kind of displacement in medio-anterio-inferior direction occurred, but it was to a lesser extent (Fig. 7IVA, IVB and Table 2). One has to remember that the sutures are not opening up in a uniform manner at all the nodes i.e., not in a parallel manner; because of this, the results are noticed in a varying pattern of negative and positive values. In this section, we are mainly concentrating on the maximum displacement which came as a positive value. The rest of the value showed negative displacement, thus, suggesting a wedge-shaped opening in this region. This appreciable displacement noted in our study is due to the fact that we have built a FE model of a 12-year-old male patient who was still left with his potential growth. However, in the earlier studies done by Gautam et al [18] and Ghonemia et al [30], non-significant difference in the width of the pterygo-maxillary suture was noted.

The maximum stresses generated in the I-RME (6.98 MPa) remained high in case of pterygo-maxillary suture as compared to the plain RME model (3.87 MPa). The stress pattern was tensile in nature for both the cases. The literature related to stress pattern for this particular suture remain scanty. The stresses generated in this suture are greater in comparison to other suture except for the midpalatal suture. As pterygo-maxillary suture is nearer to the midplatal suture, the stresses generated are greater.

### Stress and displacement at the intranasal suture

The intranasal suture in RME exhibited a displacement pattern in medio-antero-inferior direction at the posterior surface of the suture, suggesting of a wedge-shaped opening in the nasal cavity causing the widening of the same. The increase in nasal cavity width was more pronounced in the inferior portion than in the superior portion. This is in agreement with the findings of Pavlin and Vukicevic [42] who showed medial movements of the nasal process of the maxilla and other superior structures. Isère et al [19] also reported medial displacement of the posterosuperior part of the nasal cavity. The nasal cavity can widen as much as 8 to 10 mm at the level of the inferior turbinates and the nasal bone moved medially after RME [18]. In contrast to RME, I-RME showed the displacement in latero-antero-superior direction at the posterior surface of the suture. The difference in the pattern of opening may be is the site force application. The force application in case of I-RME is nearer to the intranasal suture in the vertical direction, whereas in case of the plain RME, the force application site is away from the intranasal suture.

The maximum stress generated in the RME was 0.72 and 4.26 MPa in implant-supported RME on the medial aspect of the suture (Fig. 8IA, IB and Table 2). The stress pattern remained uniformly tensile for both the cases. Our results were in agreement with the findings of earlier studies [17, 18].

### Stress and displacement at the fronto-maxillary suture

The fronto-maxillary suture showed the displacement in medio-antero-inferior direction for both the models (Fig. 8IIA, IIB and Table 2). Similar results were seen in previous studies [17, 19, 30]. However, the displacement again remained less in case of I-RME owing to the fact that the force was applied on the relatively small area of the implant. As suggested in the previous studies, the fulcrum of rotation for the two halves of maxilla remained at the fronto-maxillary suture [3, 19, 40]. However, contrasting results were reported in the study by Gautam et al [18] who found the fulcrum of rotation at the superior orbital fissure.

In previous studies of FEM on RME [17, 18], they found the increased maximum von Mises stresses at this suture, whereas in our study, the stresses generated in this suture were minimal in comparison to the midpalatal and pterygo-maxillary sutures. Maximum stresses were concentrated on the maxillary part of the fronto-maxillary suture with minimum stresses on the frontal part of the suture for both the models. Again, the stresses in the I-RME remained high in comparison to the plain RME.

### Stress and displacement at the naso-frontal suture

In RME, the naso-frontal suture displaced in medio-anterio-inferior direction but to a lesser extent. Similar results were noted in the earlier studies on RME [28, 31, 32]. In contrast, results in the previous literature showed significant increase in the naso-frontal suture width [30]. However, in I-RME, the displacement produced was in latero-antero-inferior direction.

The recorded maximum stresses were comparatively less in comparison to the other sutures for both the models (Fig. 8: IIIA-IIIB and Table 2). Nevertheless, there were reports of increased stress at the naso-frontal suture [18, 30] which were also consistent with observation on monkeys and humans [19, 40]. Maximum stresses were concentrated on the nasal part of the naso-frontal suture with minimum stresses on the frontal part of the suture in both the cases. As the suture is away from the site of application of the force, the stresses produced are also less in comparison to the other sutures.

### Stress and displacement at the zygomatico-temporal suture 

On a broad view, the zygomatico-temporal suture in RME produced a medio-antero-inferior displacement in clockwise direction. Contrastingly, in I-RME, the displacement produced was in latero-antero-inferior direction. The difference in the pattern of opening can again be attributed to the site of application of force. When the other sutures were compared to the zygomatico-temporal suture, the amount of displacement produced was negligible. Same has been stressed in the study of Gautam et al [18] who states that “The main resistance to the midpalatal suture opening is probably not in the suture itself; rather, it is in the surrounding structures with which the maxilla articulates, particularly the sphenoid and the zygomatic bones.” The same view has been shared by Isaacson and Ingram [29].

The stresses in this particular suture remained more or less same for both the models (Fig. 8: IVA-IVB and Table 2). This can be interpreted as more lateral if the structure is from the maxilla, less will be the stress generation even if it is I-RME. The dominant stress remained tensile in nature in both the models which was in contrast to the earlier reports [18, 27], in which they noticed both tensile as well as compressive stresses in the zygomatico-temporal suture.

### Stress and displacement at the zygomatico-frontal suture

In both RME and I-RME, the displacement noticed was to a lesser extent in comparison to the other sutures as seen in Table 3. The displacement was in medio-antero-inferior direction for both the models. Similar kind of displacement was noted in all the sutures except in intranasal suture which showed a superior displacement in RME model. Similar results were postulated in the study of Ghonemia et al [30] who showed insignificant increase in width of the suture. The reason behind this less displacement in fronto-zygomatic suture is increased digitation and rigidity.

The maximum stress generated in this region was 0.58 MPa with principal stress showing a compressive stress of −0.21 MPa in case of RME model. In case of the I-RME, the maximum stress generated was of 1.38 MPa and a tensile principle stress contour of 0.86 MPa (Fig. 9IA, IB and Table 2). Comparatively, these stresses were less in comparison to the stresses generated on the remaining sutures. However, contrasting results have been noted in the earlier studies [17, 19, 43].

All these varying pattern of the stresses are due to the fact that the absolute level of the induced stresses greatly depends on bone elasticity and the patient’s age. With the same orthopedic load, equivalent sutures of juvenile skulls experienced significantly higher bone strain than adult skulls, suggesting that the same mechanical force might have different biologic effects on immature and mature facial skeletons [18]. Holberg [43] demonstrated that the more nonelastic the bony structures, the higher the stresses induced on the structures of the cranial base.

### Stress and displacement at the spheno-occipital synchondrosis

The spheno-occipital synchondrosis in RME showed a medio-antero-inferior displacement with maximum displacement of 0.00002 mm in the transverse plane, 0.002 mm in the sagittal plane, and 0.0001 mm in the vertical plane. In case of the I-RME, a maximum displacement of 0.116 mm in the transverse plane, 0.580 mm in the sagittal plane, and 0.535 mm in the vertical plane indicates a medio-antero-inferior displacement pattern (Fig. 9: IIA-IIB and Table 2). However, the displacement produced is negligible when compared to the amount of displacement seen in various other sutures. This suggests that the chances of bony remodeling in the spheno-occipital synchondrosis in response to RME therapy are negligible. This supports the findings of Jafari et al [17] who noticed no displacement of the spheno-occipital synchondrosis. Contrasting reports have been noted by Gardner and Kronman [32] and Gautam et al [18] who related antero-inferior maxillary displacement to the opening of the spheno-occipital synchondrosis. The displacement pattern seen for whole of the craniofacial region is pyramidal in shape with the base in the inferior region and the apex at the superior region. The spheno-occipital synchodrosis followed the same pattern.

Maximum stresses were concentrated on the sphenoidal portion of the spheno-occipital synchondrosis with minimal stresses on the occipital portion of the spheno-occipital synchondrosis for both cases. However, in the previous study on comparison of different types of RME, procedures in adult FE model got more amount of stress at the spheno-occipital synchondrosis for both plain RME as well as surgical RME as compared to present study [43].

### Clinical significance

The speculated reason for a wide variation in the displacement pattern between two models is the junction of application of force. To elaborate, in the case of RME, the force was applied at the palatal surface of the posterior teeth, whereas in the I-RME model, the force was directly applied on the implants which were embedded in the palatal halves. Because of this, one can anticipate more amount of tipping movement in the RME model as compared to the I-RME—which is assumed to produce greater amount of orthopedic changes. Since the force applied remained same for both the models, a lesser degree of changes were seen in the I-RME model, indicating the need for an increase in the force level to obtain greater amount of displacement. The findings can be effectively utilized in choosing the type of rapid maxillary expansion appliance in day to day practice. In cases of narrow maxilla with the average to horizontal growth pattern and the palatal-tipped posteriors, the plain RME is the choice of appliance, and for vertical growing patients with narrow maxilla, implant-supported RME can be the appliance of choice, as the I-RPE appliance is anchored to the palate rather than the teeth, less dental tipping will take place, which will allow better vertical control.

### Study limitation

The validated finite element model was constructed from the CT scan data, so the thickness and morphology was accurately transformed to finite element model, thus imbibing the real life situation through the mathematical and computational model creation. However, it was assumed that all the structures of the model are to be having isotropic material properties, which is not true in real life conditions. And most of the finite element studies were carried out with this assumption, as the anisotropic data is not available. Further, the real time experiment can accurately validate the FE models. Apart from these limitations, the results are only valid for patients with comparable craniofacial structure as the reported stresses and displacement were based on the results obtained on a model that was generated from a CT scan of a 12-year-old patient.

## Conclusions

- It can be concluded that the distant structures of the craniofacial skeleton were also affected by transverse orthopedic forces.
- There was downward and forward movement of the maxilla with a tendency toward clockwise rotation in plain RME model.
- The I-RME model produced an anti-clockwise rotation of the maxilla but to a lesser extent.
- Plain RME produced increased amount of dento-alveolar tipping, whereas the I-RME produced less dento-alveolar tipping as the RME was directly anchored to the palate rather than to the tooth thus, providing the desired vertical control.
- Increased stresses were appreciated in I-RME, in contrast to that of the plain RME.
